# Outcomes and Complications of Non-operative Management of Isolated Olecranon Fractures

**DOI:** 10.7759/cureus.114026

**Published:** 2026-08-05

**Authors:** John R Vaile, Harrison Fellheimer, Jake D Fanizza, Temitope A Ayodele, Brian E Fliegel, Jonas Matzon, Justin Kistler

**Affiliations:** 1 Department of Orthopedics, Thomas Jefferson University, Philadelphia, USA; 2 Department of Orthopedic Surgery, Thomas Jefferson University, Philadelphia, USA; 3 Department of Orthopedics, Drexel University College of Medicine, Philadelphia, USA; 4 Department of Orthopedics, Zucker School of Medicine, Hempstead, USA; 5 Division of Hand Surgery, Rothman Orthopaedic Institute, Philadelphia, USA; 6 Department of Orthopedic Surgery, Jefferson Health New Jersey, Stratford, USA; 7 Department of Orthopedics, Rothman Orthopaedic Institute Foundation for Opioid Research and Education, Philadelphia, USA

**Keywords:** complication, fracture, non-operative, olecranon, outcomes

## Abstract

Introduction

Non-operative management of olecranon fractures may be indicated in specific populations or when patients opt for conservative treatment after shared decision-making. In these instances, thorough understanding of operative and non-operative management outcomes can equip upper extremity surgeons with requisite knowledge to inform practice-specific fracture management pathways. We hypothesized that non-operative management of unilateral, isolated olecranon fractures results in satisfactory patient outcomes in terms of function, range of motion, and pain.

Methods

After International Review Board (IRB) approval, retrospective chart review was conducted on adults (>18 years) with unilateral, isolated olecranon fractures at a single multispecialty orthopedic practice from 2010 to 2024. Exclusion criteria included polytrauma and open injuries. Demographics, injury characteristics, fracture care management, complications, and functional outcomes were collected. Descriptive statistics were performed using SPSS version 28 (IBM, Armonk, NY).

Results

In 163 patients (mean age 73.2 ± 20.4), non-displaced (n = 57) and displaced (n = 106) fractures had similar range of motion (ROM): flexion 132° vs. 130° (p = 0.099), extension 11.1° vs. 11.3° (p = 0.523), supination 78.5° vs. 77.8° (p = 0.132); pronation was slightly higher in non-displaced (79.5° vs. 77.8°, p = 0.048). Complications were comparable (12.0% vs. 11.6%, p = 1.000); conversion to surgery was rare (1.79% vs. 0.94%, p = 1.000). Age-stratified analysis (<50, 50-59, 60-69, 70-79, 80-89, 90+) showed declining ROM with age: elbow flexion from 135° (<50) to 100° (90+) and extension from 6.05° (<50) to 15.9° (90+).

Conclusion

Non-operatively managed unilateral, isolated olecranon fractures demonstrated near-full elbow ROM at final follow-up visit. On average, ROM was comparable between displaced and non-displaced fractures, with no significant differences in flexion, extension, or supination; pronation was slightly reduced in displaced fractures (77.8° vs. 79.5°, p = 0.048). Overall complication rate was low (11.7%), and conversion to surgery was rare (1.23%), with no significant differences between displaced and non-displaced groups. Age-stratified analysis revealed statistically significant declining ROM and increasing complication and surgical conversion rates with increased age. This study reports the largest cohort of adult patients treated non-operatively for unilateral, isolated olecranon fractures, with age-stratified outcome data.

## Introduction

Olecranon fractures account for approximately 10% of elbow fractures in adults [1-3]. High-impact trauma, such as motor vehicle accidents, is the leading cause of injury in younger males, whereas low-impact falls are more common among elderly females [4,5]. Surgical management, particularly open reduction and internal fixation (ORIF), remains the traditional treatment for displaced olecranon fractures due to high union rates and favorable outcomes [6-8]. However, complications, such as infection, hardware irritation, and reoperation, are not uncommon [7,9].

In recent years, non-operative treatment, typically involving short-term immobilization followed by physical therapy, for olecranon fractures has gained traction. This can be advantageous for older adults with non-displaced fractures, low functional demands, or significant comorbidities [10-12]. Over the last 20 years, the rate of non-operative management for these injuries has risen with a particularly notable increase in patients over the age of 75 [12]. Prior studies have characterized the clinical outcomes for conservative treatment in selected populations, particularly those aged 65 and older, showing good range of motion (ROM), low reported pain, and high patient satisfaction [11,13]. Despite this, there remains a lack of high-powered evidence assessing functional and pain outcomes in large, non-operatively managed cohorts, and debate persists regarding optimal immobilization duration [14,15].

The purpose of our current study is to evaluate the outcomes and complications of non-operative management of unilateral, isolated olecranon fractures in relation to ROM and self-reported pain scores at a single multispecialty orthopedic practice.

## Materials and methods

After International Review Board (IRB) approval, a patient cohort was queried from an internal database of a large multispecialty academic orthopedic practice. Patients were identified using International Classification of Diseases 10th Revision (ICD-10) codes for olecranon fractures (S52.021, S52.022, S52.023, S52.024, S52.025, S52.026, S52.031, S52.032, S52.033, S52.034, S52.035, S52.036) and Current Procedural Terminology (CPT) codes for closed treatment of olecranon fractures (24670, 24675). All adult patients (≥18 years old) with isolated, unilateral olecranon fractures treated non-operatively were included. Exclusion criteria were patients with initial surgical management, polytrauma, or open injuries. Patients with incomplete follow-up information and patients without an initially described displacement classification of displaced or non-displaced were excluded from final statistical analysis. The primary outcome was ROM at final follow-up. Secondary outcomes were Disabilities of the Arm, Shoulder, and Hand (DASH) Scores for functionality, total time splinted or casted, and total length of follow-up.

Each patient’s electronic medical record and plain radiographs were reviewed to categorize fracture cohorts. Displaced and non-displaced categorization was determined by chart review done by an orthopedic resident. Fracture laterality and hand dominance were also recorded.

Quantitative ROM for elbow extension, elbow flexion, forearm supination, and forearm pronation was collected from clinic notes at initial and final follow-up visits. For notes with a descriptive ROM, “full ROM” was converted to 0° extension, 140° flexion, 80° supination, and 80° pronation, which are based on consensus values from the literature [16]. For patients lacking degrees of motion in a particular plane, this value was subtracted from the consensus value to calculate the ROM in a given plane. For example, “lacking 15° of flexion” per clinic note was recorded as 125° of elbow flexion.

Time spent splinted or casted and total follow-up duration were recorded, with immobilization time defined as the period from injury occurrence until mobilization. All complications, fracture care methods, conversions to surgery, and total time to conversion were recorded.

Statistical analysis

Statistical analysis was performed across the non-operative cohort and age-based subgroups for both displaced and non-displaced fracture cohorts. Demographics, laterality, hand dominance, displacement, complications, and conversion to surgery were calculated as proportions (percentages) of the overall cohort. Mean and standard deviation (SD) were calculated for ROM measurements. T-tests or Mann-Whitney U tests were used to compare ROM, length of follow-up, and time splinted/casted across two age subgroups. ANOVA or Kruskal-Wallis tests were used across age subgroups of three or more. Chi-square tests were used to compare categorical data between displaced and non-displaced groups. When appropriate, multiple comparison tests were performed. Five patients were not categorized as non-displaced or displaced.

## Results

A total of 168 patients were included in this study, with 163 of these patients divided into two subgroups: non-displaced fractures (n = 57) and displaced fractures (n = 106). The remaining five patients could not be categorized into displaced or non-displaced groupings and were excluded from analysis. Of the total sample, 114 were female (67.9%), and 54 were male (32.1%). The non-displaced cohort consisted of 40 (70.2%) female patients, and the displaced cohort had 69 (65.1%) female patients. The mean patient age was 73.2 years with an SD of 20.4. A significant age difference was observed between the groups, with the non-displaced group having a younger mean age of 62.5 years (SD 24.1) compared to 79.0 years (SD 15.4) in the displaced group (p < 0.001) (Table 1).

**Table 1:** Patient demographics Summary of patient characteristics including sex, age, procedure arm, and handedness. p-values denote comparison of non-displaced versus displaced cohorts. Five patients were not classified as non-displaced or displaced and were excluded from overall analysis.

Patient demographics	Total	Non-displaced	Displaced	p-value	Statistical test	Statistics
	N = 163	N = 57	N = 106	-	-	-
Female	109 (66.9%)	40 (70.2%)	69 (65.1%)	0.629	Chi-square test	0.23
Male	54 (33.1%)	17 (29.8%)	37 (34.9%)			
Mean age	73.2 (20.4)	62.5 (24.1)	79.0 (15.4)	<0.001	Chi-square test	29.44
Age ≤ 50 years	24 (14.7%)	18 (31.6%)	6 (5.66%)	<0.001	Mann-Whitney U test	1798.5
Age 50-59 years	12 (7.36%)	7 (12.3%)	5 (4.72%)			
Age 60-69 years	20 (12.3%)	5 (8.77%)	15 (14.2%)			
Age 70-79 years	31 (19.0%)	12 (21.1%)	19 (17.9%)			
Age 80-89 years	40 (24.5%)	6 (10.5%)	34 (32.1%)			
Age ≥ 90 years	36 (22.1%)	9 (15.8%)	27 (25.5%)			
Laterality: left	88 (54.0%)	33 (57.9%)	55 (51.9%)	0.569	Chi-square test	0.32
Laterality: right	75 (46.0%)	24 (42.1%)	51 (48.1%)			
Hand dominance: left	13 (10.6%)	3 (7.14%)	10 (12.3%)	0.326	Fisher's exact test	NA
Hand dominance: right	109 (88.6%)	38 (90.5%)	71 (87.7%)			
Hand dominance: ambidextrous	1 (0.81%)	1 (2.38%)	0 (0.00%)			

Table 2 highlights the mean differences in ROM between displaced and non-displaced groups. Apart from forearm pronation (non-displaced: 79.5° (SD 5.04); displaced: 77.8° (SD 9.18) (p = 0.048), no significant differences in ROM were seen.

**Table 2:** Overall outcomes by patient characteristics Summarizes ROM and treatment outcomes of all patients. p-values denote comparison of non-displaced versus displaced cohorts. Five patients were not classified as non-displaced or displaced and were excluded from overall analysis. DASH, Disabilities of the Arm, Shoulder, and Hand; ROM, range of motion

Outcome measures	Total	Non-displaced	Displaced	p-value	Statistical test	Statistics
	N = 163	N = 57	N = 106	-	-	-
Degrees elbow extension	11.2 (15.5)	11.1 (14.7)	11.3 (16.1)	0.523	Mann-Whitney U test	1804
Degrees elbow flexion	131 (14.5)	132 (15.9)	130 (13.7)	0.099	Mann-Whitney U test	2255.5
Degrees forearm supination	78.1 (8.87)	78.5 (8.41)	77.8 (9.17)	0.132	Mann-Whitney U test	1541.5
Degrees forearm pronation	78.4 (7.96)	79.5 (5.04)	77.8 (9.18)	0.048	Mann-Whitney U test	1555
DASH score	20.1 (18.9)	18.5 (22.8)	25.0 (.)	-	-	-
Complications	17 (11.7%)	6 (12.0%)	11 (11.6%)	1	Chi-square test	0
Surgical conversions	2 (1.23%)	1 (1.79%)	1 (0.94%)	1	Fisher's exact test	NA
Days immobilized	26.6 (16.3)	22.6 (14.5)	28.8 (17.0)	0.079	Mann-Whitney U test	1454.50

Table 3 summarizes surgical conversion, immobilization, and complications, such as nonunion or malunion, loss of extension strength, persistent pain, ulnar nerve irritation, joint stiffness, and post-traumatic arthritis. Ultimately, complications occurred in 11.7% of cases, with similar rates between the non-displaced (12.0%) and the displaced group (11.6%) (p = 1.000). Conversion from non-surgical to surgical management was rare, occurring in only 1.23% of cases overall. Specifically, 1.79% of the non-displaced group and 0.94% of the displaced group required surgical intervention (p = 1.000). Mean duration (days) of immobilization was 26.6 (SD 16.3) across the cohort. The non-displaced group had a shorter mean time of 22.6 days (SD 14.5) compared to 28.8 days (SD 17.0) in the displaced group (p = 0.079). Overall, surgical conversion was rare, with the two interventions seen in patients over 80.

**Table 3:** Outcomes and characteristics by age Displays patient characteristics and outcomes by age at time of injury. For each age subgroup, the percentage of patients with the characteristic or SD is denoted by parentheses. DASH scores were not present in every subgroup. The outcomes of each age subgroup were compared to other subgroups. DASH, Disabilities of the Arm, Shoulder, and Hand

Characteristics/outcomes	Total	Age ≤ 50 years	Age 50-59 years	Age 60-69 years	Age 70-79 years	Age 80-89 years	Age ≥ 90 years
	N = 168	N = 24	N = 13	N = 21	N = 32	N = 42	N = 36
Female	114 (67.9%)	10 (41.7%)	8 (61.5%)	13 (61.9%)	20 (62.5%)	34 (81.0%)	29 (80.6%)
Male	54 (32.1%)	14 (58.3%)	5 (38.5%)	8 (38.1%)	12 (37.5%)	8 (19.0%)	7 (19.4%)
Mean age	73.2 (20.2)	33.9 (11.0)	55.5 (2.73)	65.8 (2.34)	75.6 (2.76)	85.4 (2.86)	93.8 (3.16)
Laterality: left	88 (53.3%)	17 (70.8%)	8 (66.7%)	8 (40.0%)	19 (59.4%)	19 (46.3%)	17 (47.2%)
Laterality: right	77 (46.7%)	7 (29.2%)	4 (33.3%)	12 (60.0%)	13 (40.6%)	22 (53.7%)	19 (52.8%)
Hand dominance: left	13 (10.6%)	1 (5.00%)	1 (14.3%)	1 (6.25%)	1 (4.35%)	5 (15.6%)	4 (16.0%)
Hand dominance: right	109 (88.6%)	19 (95.0%)	6 (85.7%)	15 (93.8%)	21 (91.3%)	27 (84.4%)	21 (84.0%)
Hand dominance: ambidextrous	1 (0.81%)	0 (0.00%)	0 (0.00%)	0 (0.00%)	1 (4.35%)	0 (0.00%)	0 (0.00%)
Displacement	106 (65.0%)	6 (25.0%)	5 (41.7%)	15 (75.0%)	19 (61.3%)	34 (85.0%)	27 (75.0%)
Elbow extension	11.1 (15.5)	6.05 (10.8)	10.0 (11.0)	4.47 (6.97)	14.3 (17.7)	11.7 (17.8)	15.9 (16.9)
Elbow flexion	131 (14.4)	135 (10.2)	134 (12.6)	135 (9.00)	128 (17.8)	133 (11.4)	124 (18.4)
Forearm supination	78.1 (8.83)	79.5 (2.76)	80.0 (0.00)	76.2 (9.61)	74.8 (12.8)	78.7 (5.93)	80.6 (11.1)
Forearm pronation	78.4 (7.92)	79.1 (4.02)	80.0 (0.00)	76.2 (9.61)	76.5 (9.82)	78.7 (5.93)	80.6 (11.1)
DASH score	20.1 (18.9)	11.4 (0)	-	34.5 (13.4)	0.00 (0)	-	-
Complications	17 (11.6%)	1 (5.56%)	1 (9.09%)	0 (0.00%)	6 (20.7%)	5 (13.2%)	4 (12.5%)
Surgical conversion	2 (1.20%)	0 (0.00%)	0 (0.00%)	0 (0.00%)	0 (0.00%)	1 (2.38%)	1 (2.86%)
Days immobilized	26.6 (16.3)	16.9 (10.3)	27.4 (21.0)	20.3 (13.7)	27.9 (19.9)	31.5 (17.9)	28.6 (11.7)

## Discussion

Selection of treatment for olecranon fractures must account for individual patient factors, including age, comorbidities, functional demand, and patient preference [17]. While surgical management often remains the traditional treatment for displaced fractures [18], our findings underscore the utility of non-operative management, particularly in older adults or patients with significant surgical risk. Non-operative management may be preferred for those with low functional demand, significant comorbidities, or personal preference against surgery. Furthermore, surgeons often stratify risk and recommend conservative treatment for patients unlikely to tolerate surgery or benefit significantly from its outcomes. These considerations highlight the importance of a personalized approach to treatment.

 Our current study shows that non-operative management yielded satisfactory results, with a 11.7% complication rate. Functional ROM (generally considered to be 30° of extension to 130° of flexion) was preserved in most patients, with mean elbow extension and flexion across the cohort demonstrating functional values. However, patients aged 70-79 and aged 90+ had less than functional flexion with 128° and 124°, respectively. These findings are further supported by Alvara et al., who reported satisfactory ROM and complication outcomes in patients older than 70 with olecranon fractures treated non-operatively [19]. These results suggest that non-operative treatment is a viable option for unilateral isolated olecranon fractures and is especially relevant in cases where surgical risks outweigh potential benefits. The rare need for surgical conversion further supports the durability of conservative management in selected patients.

Furthermore, compared to prior literature, our findings highlight key distinctions in outcomes, based on both age and fracture displacement status. Younger patients (age<75) exhibited greater ROM and lower complication rates across all planes of elbow motion, with a progressive decline seen with advancing patient age. Specifically, flexion decreased from a mean of 135° in patients <50 to 124°in those over 90, and extension from 6.05° in patients <50 to 15.9° in patients over 90. Supination and pronation similarly had a limited trend with age. Complication rates mimicked a non-normalized bell-shaped trend, peaking in the 70-79 age group (6; 20.7%), before decreasing again in older aged patients. Interestingly, while displaced fractures were more common in older patients, functional outcomes - including ROM and pain - remained largely preserved, and complication rates were not significantly higher than non-displaced fractures. These findings are consistent with prior studies, which reported that elderly patients with displaced olecranon fractures can achieve satisfactory outcomes with non-operative treatment [11,13]. Our results further support this argument by showing that, even in the presence of fracture displacement and advanced age, non-operative management can yield acceptable pain and motion outcomes with minimal need for surgical escalation. This finding underscores the potential value of conservative management in elderly populations, thereby challenging the assumption that fracture displacement always necessitates operative fixation in this demographic.

Older patients tended to report higher pain levels, potentially reflecting differences in fracture displacement, medical comorbidities, or baseline pain thresholds. Despite this, the overall pain outcomes remained acceptable, with most patients achieving substantial pain relief by final follow-up. However, it should be noted that patients’ perception of what pain level is acceptable or tolerable is subjective and remains difficult to quantify. Notably, the increased prevalence of displaced fractures in older patients did not significantly worsen complication rates or necessitate surgical intervention; perhaps careful fracture management and patient selection can mitigate the risks associated with more complex injuries.

Lastly, the duration of immobilization also varied by age, with older patients requiring slightly longer splinting times. This finding may reflect the increased need for stability in osteoporotic bone or the tendency toward conservative post-fracture protocols in older adults. However, it is important to understand that prolonged immobilization may contribute to stiffness. Patients in our cohort were splinted for a duration of approximately three or four weeks. However, to our knowledge, there is no definitive data to determine the appropriate duration of splinting or immobilization. We recommend splinting and immobilization until the patient is comfortable and the soft tissue envelope is amenable to early active ROM. With this in mind, it may be reasonable to allow early ROM and daily activities within the first week following injury, but the validity of this must be explored in future analyses. These data demonstrated an average immobilization time of 22.6 days for non-displaced and 28.8 days for displaced fractures. Perhaps splinting beyond four weeks may not confer additional clinical benefit and instead may contribute to increased complications (e.g., stiffness) in patients with advanced age.

This study has several limitations. First, the retrospective design introduces selection and information bias. The study’s reliance on clinical records for outcome data may have led to incomplete documentation of functional measures, such as ROM or pain scores, particularly in patients lost to follow-up. This limited our ability to thoroughly analyze functional outcomes using DASH Scores, which were continuously reported for only 20 patients. Another limitation of this study is the reduced precision of range-of-motion data, as qualitative office note descriptors such as “full ROM” were converted into quantitative measurements. However, to our knowledge, this study still captures the largest cohort of non-operatively treated olecranon fractures to date. Second, while we focused exclusively on non-operative fracture management, there remains a lack of direct comparison to fractures treated surgically. Therefore, it is difficult to directly compare the relative efficacy of each approach. Third, the non-operative cohort’s absence of standardized immobilization protocols introduces variability that could affect outcomes. Fourth, while our multi-provider practice has variability in treatment management pathways, the single system may limit the generalizability of our findings to other practices with differing practice protocols. Fifth, the results of our study may not be generalizable to all patients and populations. However, our large practice is spread across a large geographical area encompassing multiple socioeconomic populations, and we would argue that this does add strength to our results. Future research, including prospective studies or randomized controlled trials, is necessary to address these limitations.

## Conclusions

In conclusion, non-operative management of unilateral isolated olecranon fractures can be considered across a range of patient demographics, though outcomes are affected by age and fracture displacement. Younger patients generally achieve better ROM and lower complication rates, while older patients benefit from conservative treatment that avoids surgical risks. Surgeons should weigh patient-specific factors, such as personal preference, age, comorbidities, and functional demands, when formulating treatment plans. Future studies should aim to refine treatment algorithms, explore alternative immobilization durations, and further evaluate long-term functional outcomes to improve care for this common injury.
